# Immune Checkpoint Inhibitor-Associated Pneumonitis in Patients Treated for Metastatic Renal Cell Carcinoma

**DOI:** 10.3390/curroncol33100616

**Published:** 2026-10-14

**Authors:** Jack Masur, Abhishek Mullapudi, Soham Ali, Kathryn Fortune, Juliana Bueno, Thomas Battey, Ariaz Goudarzi, Hitesh Patel, Jeffrey Sturek, Michael Devitt, William Paul Skelton, Robert Dreicer, Paul Viscuse

**Affiliations:** 1 Division of Hematology/Oncology, Department of Internal Medicine, University of Virginia, Charlottesville, VA 22903, USA; 2 Department of Internal Medicine, University of Virginia School of Medicine, Charlottesville, VA 22903, USA; 3 Department of Internal Medicine, University of Virginia, Charlottesville, VA 22903, USA; 4 Department of Radiology and Medical Imaging, University of Virginia, Charlottesville, VA 22903, USA; 5 Division of Pulmonary and Critical Care Medicine, Department of Internal Medicine, University of Virginia, Charlottesville, VA 22903, USA

**Keywords:** renal cell carcinoma, immune checkpoint inhibitor, immunotherapy, immune-related adverse event, pneumonitis, treatment-related toxicity

## Abstract

Immune checkpoint inhibitors (ICIs) improve outcomes in metastatic renal cell carcinoma (mRCC) but may be associated with pneumonitis. We retrospectively studied adults with mRCC who began first- or second-line ICI therapy at the University of Virginia Medical Center between 2015 and 2023. Potential cases underwent multidisciplinary clinical review, and two thoracic radiologists independently reviewed available computed tomography scans. Among 146 ICI-treated patients, 13 developed pneumonitis (8.9%; 95% CI 4.8–14.7%). Incidence did not differ significantly by treatment line or ICI regimen. Incidence was higher with prior chest radiation than without (31.8% vs. 4.8%; unadjusted odds ratio 9.2, 95% CI 2.7–31.0; Benjamini–Hochberg-adjusted *p* = 0.005). Five cases (38%) were grade 3–4 at diagnosis, and seven (54%) reached grade ≥ 3. Nine patients (69%) received high-dose systemic corticosteroids. Eleven patients (85%) improved or resolved with initial management; two, both with grade 1–2 pneumonitis at diagnosis, worsened and died (grade 5). Five patients underwent ICI rechallenge after clinical recovery, without recurrent pneumonitis. In this exploratory cohort, pneumonitis was sometimes fatal despite a low grade at presentation. The association with prior chest radiation is hypothesis-generating and warrants evaluation in larger studies with detailed radiation and confounder data.

## Simple Summary

Immunotherapy has improved outcomes for many people with metastatic kidney cancer, but it can cause inflammation of the lungs, known as pneumonitis. This complication can be difficult to distinguish from infection, cancer progression, or prior radiation-related lung injury and can sometimes be life-threatening. We retrospectively reviewed 146 patients with metastatic kidney cancer treated with immunotherapy at a single academic institution. Pneumonitis occurred in 13 (8.9%) of these patients. It was observed more often among patients who had previously received radiation to the lung or mediastinum, but this study could not account for other factors, so this observation needs confirmation. Most patients improved with treatment. Pneumonitis was severe in more than half of affected patients, and two patients died, although both first presented with mild or moderate pneumonitis. Five patients restarted immunotherapy after recovery without recurrent pneumonitis. These findings support close monitoring for respiratory symptoms during and after immunotherapy.

## 1. Introduction

An estimated 80,450 people will be diagnosed with cancers of the kidney and renal pelvis in the United States in 2026, and approximately 15,160 will die of their disease [1]. Renal cell carcinoma (RCC) is the most common malignant tumor of the kidney, accounting for approximately 85% of all renal malignancies [2]. Around 20–30% of patients with RCC present with metastatic disease at diagnosis [3]. An additional 20–30% of patients with localized tumors at diagnosis experience recurrence after surgical excision, with the lung being the most common site, representing 50–60% of cases [4].

Metastatic RCC (mRCC) often requires systemic therapy to prolong survival [2]. Immune checkpoint inhibitors (ICIs) have become standard first-line treatment for most patients with mRCC based on phase III trials demonstrating improved overall survival with ICI-containing regimens compared with vascular endothelial growth factor tyrosine kinase inhibitor (TKI) monotherapy [5,6,7,8]. In a Dutch nationwide cohort of patients with synchronous mRCC, 94% of those receiving first-line systemic therapy in 2018 were treated with a TKI, whereas 79% received ICI-based therapy by 2022 [9].

As ICI use has expanded, so has the risk of immune-related adverse events (irAEs). Pneumonitis is an uncommon but potentially serious pulmonary complication that can be challenging to distinguish from infection, tumor progression, radiation-associated injury, and other pulmonary conditions. Pneumonitis attributed to ICI therapy can cause substantial morbidity and, in severe cases, death [10,11]. However, disease-specific data describing pneumonitis among patients with mRCC treated with ICI are limited.

Most estimates of pneumonitis risk in RCC come from registration trials and trial-level meta-analyses [12,13,14], whose eligibility criteria exclude many patients treated in practice, or from real-world cohorts spanning several tumor types in which lung cancer predominates [15]. Findings from lung cancer may not transfer to RCC. In RCC, the lung is affected by metastases rather than by the primary tumor, and pulmonary metastases are common [16]. In addition, ipilimumab plus nivolumab, which ranked highest for pneumonitis among first-line regimens in a network meta-analysis [14], is widely used. Diagnosis is also difficult in RCC, where respiratory symptoms and computed tomography (CT) findings overlap with pulmonary metastases, infection, heart failure, and TKI-related lung toxicity [16]. We identified few RCC-specific series that combine adjudicated case identification with radiologic characterization, which motivated this review of our institutional experience.

This single-center retrospective cohort study was designed to describe the incidence, clinical features, management, radiologic features, and outcomes of pneumonitis occurring during or after ICI treatment in patients with mRCC. Secondary exploratory objectives were to evaluate associations between selected baseline clinical characteristics, treatment exposures, and pneumonitis incidence.

## 2. Materials and Methods

### 2.1. Study Design and Population

This was a single-center retrospective cohort study conducted at the University of Virginia Medical Center. Eligible patients were adults aged 18 years or older with pathologically confirmed RCC, radiographic evidence of metastatic disease, and initiation of first- or second-line ICI therapy between 2015 and 2023. Clinical and demographic data included age, sex, race, Eastern Cooperative Oncology Group performance status (ECOG PS), date of mRCC diagnosis, histology, metastatic site(s), line and type of ICI regimen, treatment duration, smoking status, pulmonary comorbidities, prior nephrectomy, receipt of prior chest radiation, and baseline laboratory values. Regimens were grouped as ICI/ICI (ipilimumab plus nivolumab), ICI/TKI, or ICI monotherapy. Prior chest radiation was defined as radiotherapy to the lung or mediastinum completed before ICI initiation.

International Metastatic RCC Database Consortium (IMDC) risk at ICI initiation was calculated from the six factors described by Heng et al. [17]: less than 1 year from diagnosis to systemic therapy; Karnofsky performance status below 80%, with ECOG PS of 2 or higher used as a surrogate; hemoglobin below the lower limit of normal (13.5 g/dL for men and 12.0 g/dL for women); calcium above 10.2 mg/dL; neutrophil count above 7000/µL; and platelet count above 400 × 10^3^/µL. Albumin was not available, so uncorrected calcium was used. Patients were classified as favorable (no factors), intermediate (one or two factors), or poor risk (three or more factors); patients with missing factors that could change the category were classified as unknown.

### 2.2. Pneumonitis Case Identification and Clinical Review

Potential pneumonitis cases were identified through full review of the medical records of all 146 patients and evaluated using contemporaneous clinical documentation and available thoracic imaging. Cases were included when the treating team and multidisciplinary review considered pneumonitis to be the most likely diagnosis in the setting of ICI exposure, with no upper limit on the interval after the last ICI dose. Patients with a documented alternative primary etiology, including active pulmonary infection or malignant pulmonary infiltration, were to be excluded; no suspected case met this criterion. When the record documented a possible alternative or contributing cause, it was abstracted and is reported in Table S1. Because this was a retrospective study, diagnostic work-up was not standardized across patients, and no patient underwent bronchoscopy.

For included cases, clinical and radiologic features were assessed by a multidisciplinary team of medical oncologists, pulmonologists, and radiologists. Pneumonitis was graded according to the Common Terminology Criteria for Adverse Events (CTCAE), version 5.0; grade was assigned retrospectively by the study team from contemporaneous documentation of symptoms, oxygen requirement, level of care, and interventions. Both the grade at diagnosis and the highest grade reached were recorded, and death to which pneumonitis contributed was classified as grade 5, with attribution recorded as primary or contributory. Corticosteroid route and starting dose were recorded; high-dose corticosteroids were defined as 1 mg/kg/day or more of prednisone equivalent. Steroid-refractory pneumonitis was defined as no clinical improvement after 48 h of high-dose corticosteroids [18]. Recurrence with or without ICI rechallenge was recorded, and the interval from the last ICI dose to pneumonitis onset was calculated.

Receipt of prior chest radiation was abstracted from the medical record before ICI initiation. Radiation dose, fractionation, field, dose-volume parameters, and the interval between radiation and ICI initiation were not available for analysis. Accordingly, radiation fields were not considered in the radiologic review, and the study could not determine whether radiographic abnormalities overlapped prior radiation fields.

### 2.3. Radiologic Review

Available computed tomography scans from patients with pneumonitis were independently reviewed by two thoracic radiologists blinded to clinical data, followed by consensus review when necessary. Readers did not have access to radiation treatment plans. Radiologic features were classified as cryptogenic organizing pneumonia (COP)-like, ground-glass opacities (GGO), hypersensitivity, interstitial, or pneumonitis not otherwise specified (NOS) [10,19]. Radiologic severity was classified as mild, moderate, or severe. Baseline thoracic metastatic burden was evaluated qualitatively by number of lung target lesions and quantitatively by the lung target-lesion sum of measurements (iSOM, in millimeters). Best treatment response was evaluated using the immune Response Evaluation Criteria in Solid Tumors (iRECIST) [20]. Metastatic burden, pneumonitis severity and outcome, and best treatment response were determined from serial chest computed tomography scans when available.

### 2.4. Statistical Analysis

Descriptive statistics summarized patient characteristics and outcomes; quartiles were calculated with the weighted average method. Incidence is reported with Clopper–Pearson exact 95% confidence intervals (CIs). Associations between pneumonitis incidence and selected patient characteristics or treatment exposures were assessed with Fisher’s exact test for two-group comparisons and the Fisher–Freeman–Halton exact test for comparisons across three or more groups. *p* values for the seven reported comparisons (treatment line, ICI regimen, metastatic distribution, prior chest radiation, cytoreductive nephrectomy, underlying lung disease, and smoking history) were adjusted using the Benjamini–Hochberg method. The odds ratio for prior chest radiation is reported with a Woolf 95% CI. Given the small number of pneumonitis events (*n* = 13), multivariable modeling was not performed to avoid model overfitting. Incidence by treatment era (ICI initiation before vs. on or after 16 April 2018, the date of U.S. Food and Drug Administration approval of first-line nivolumab plus ipilimumab for intermediate- or poor-risk advanced RCC) was compared post hoc and is reported without adjustment. The cumulative incidence of pneumonitis from ICI initiation was estimated with the Aalen–Johansen method, with death without pneumonitis treated as a competing risk; one patient without follow-up time was excluded from this analysis. In a sensitivity analysis, the three cases with onset more than 90 days after the last ICI dose were reclassified as non-cases [21]. Missing data were not imputed; analyses used available data for each variable. Imaging availability for radiologic characterization is reported descriptively. Analyses were performed in Python 3.11 with SciPy 1.17.

## 3. Results

### 3.1. Patient Characteristics

Data were available for 146 patients with mRCC who began first- or second-line ICI therapy between 2015 and 2023; 127 (87%) started ICI on or after 16 April 2018. Of these, 117 patients (80%) received first-line treatment and 29 (20%) received second-line treatment. Most patients received dual ICI/ICI therapy (83/146, 57%), followed by ICI monotherapy (43/146, 29%) and ICI/TKI therapy (20/146, 14%). The median age at ICI initiation was 63.5 years (interquartile range, 55–70 years). Overall, 108 patients (74%) were male, 133 (91%) were White, and 107 (73%) had pure clear cell histology. Metastatic sites at mRCC diagnosis included lung only in 17 patients (12%), lung plus visceral organ or bone in 48 (33%), lung plus soft tissue or lymph node in 54 (37%), and sites other than lung in 27 (18%). Current or former smoking was reported in 83 patients (57%), underlying lung disease in 26 (18%), prior cytoreductive nephrectomy in 82 (56%), and prior chest radiation in 22 (15%). By IMDC criteria, 7 patients (5%) were favorable risk, 84 (58%) intermediate risk, and 28 (19%) poor risk; 27 (18%) could not be classified because baseline laboratory values were missing. Additional baseline characteristics are shown in Table 1.

### 3.2. Incidence of Pneumonitis

Pneumonitis was identified in 13 of 146 patients (8.9%; 95% CI 4.8–14.7%). It occurred in 9.4% (11/117) of patients treated in the first-line setting and 6.9% (2/29) of patients treated in the second-line setting (adjusted *p* > 0.99). Incidence was 10.8% (9/83) among patients receiving ICI/ICI therapy, 10.0% (2/20) among those receiving ICI/TKI therapy, and 4.7% (2/43) among those receiving ICI monotherapy (adjusted *p* > 0.99). Incidence was 11.8% (2/17) in patients with lung-only metastases, 9.3% (5/54) in those with lung plus soft tissue/nodal metastases, 8.3% (4/48) in those with lung plus visceral/bone metastases, and 7.4% (2/27) in those with metastases outside the lung (adjusted *p* > 0.99).

**Figure 1:**
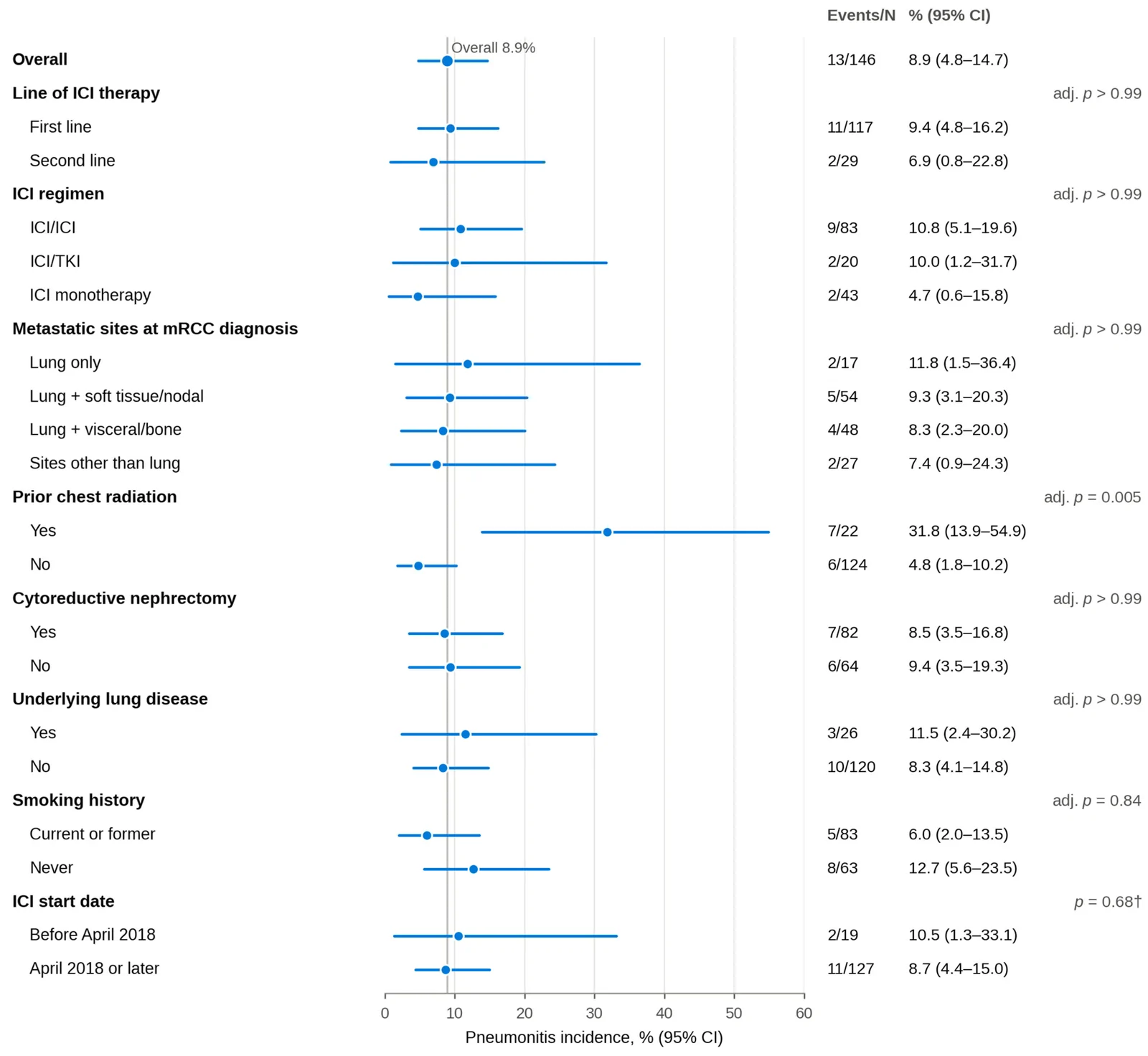
Incidence of pneumonitis by clinical characteristics. Points show incidence and horizontal lines show exact 95% confidence intervals; the vertical line marks the overall incidence of 8.9% (13/146). Rates were similar between first-line (11/117; 9.4%) and second-line (2/29; 6.9%) therapy. Pneumonitis occurred in 10.8% of patients receiving ICI/ICI, 10.0% with ICI/tyrosine kinase inhibitor (TKI), and 4.7% with ICI monotherapy. Pneumonitis incidence was higher among patients with prior chest radiation than among those without (31.8% vs. 4.8%; adjusted *p* = 0.005); this univariate, exploratory association does not account for radiation dose, field, timing, or thoracic tumor burden. Benjamini–Hochberg-adjusted *p* values across seven comparisons are shown for each characteristic; the treatment-era comparison (†) was added post hoc and is unadjusted. ICI, immune checkpoint inhibitor; TKI, tyrosine kinase inhibitor.

In exploratory univariate analysis, pneumonitis incidence was higher among patients with prior chest radiation than among those without prior chest radiation (7/22 [31.8%; 95% CI 13.9–54.9%] vs. 6/124 [4.8%; 95% CI 1.8–10.2%]; odds ratio 9.2, 95% CI 2.7–31.0; adjusted *p* = 0.005; Figure 1). Pneumonitis incidence did not differ significantly by underlying lung disease (11.5% vs. 8.3%; adjusted *p* > 0.99), smoking status (6.0% among current/former smokers vs. 12.7% among never smokers; adjusted *p* = 0.84), or prior cytoreductive nephrectomy (8.5% vs. 9.4%; adjusted *p* > 0.99). Incidence was 10.5% (2/19) among patients who started ICI before 16 April 2018 and 8.7% (11/127) among those who started on or after that date (*p* = 0.68). The cumulative incidence of pneumonitis 12 months after ICI initiation was 25.1% among patients with prior chest radiation and 5.2% among those without (Figure S1); 11 of 22 patients with prior chest radiation remained under observation at 12 months. After reclassification of the three cases with onset more than 90 days after the last ICI dose, incidence was 6.8% (10/146), and the difference by prior chest radiation persisted (5/22 vs. 5/124; unadjusted *p* = 0.008).

### 3.3. Clinical Features of Pneumonitis

The onset of pneumonitis ranged from 21 to 756 days after ICI initiation, with a median onset of 73 days (interquartile range, 47–256 days). Median onset was 245 days (range, 47–756) among patients with prior chest radiation and 45 days (range, 21–273) among those without. Six cases began after ICI had been discontinued, three of them more than 90 days after the last dose (140, 252, and 735 days). At diagnosis, four patients (30.8%) had grade 1 pneumonitis, four (30.8%) had grade 2, four (30.8%) had grade 3, and one (7.7%) had grade 4 pneumonitis. The highest grade reached was grade 1 in three patients, grade 2 in three, grade 3 in four, grade 4 in one, and grade 5 in two; seven patients (53.8%) reached grade 3 or higher. The most common presenting symptoms were cough (8/13, 61.5%), dyspnea (7/13, 53.8%), and fever (3/13, 23.1%). Five patients (38.5%) required new oxygen therapy at diagnosis, and three (23.1%) were asymptomatic. Five patients (38.5%) experienced additional irAEs, including rash (*n* = 3), endocrinopathy (*n* = 3), arthritis (*n* = 2), colitis (*n* = 1), and encephalitis (*n* = 1). The treating team documented a possible alternative or contributing cause in seven cases: infection (*n* = 4), radiation pneumonitis (*n* = 2), heart failure or volume overload (*n* = 2), and treatment effect on tumor (*n* = 1) (Table S1).

### 3.4. Radiologic Features of Pneumonitis

Available imaging was reviewed in 11 of 13 pneumonitis cases. Among four patients with grade 1 pneumonitis, two (50%) had a cryptogenic organizing pneumonia-like phenotype, one (25%) had a ground-glass opacity phenotype, and one (25%) had a hypersensitivity phenotype. Radiologic severity was mild in two patients, moderate in one, and severe in one. Among four patients with grade 2 pneumonitis, one had a hypersensitivity phenotype, one had pneumonitis not otherwise specified, and two did not have imaging available for review because the diagnosis was made clinically. Among four patients with grade 3 pneumonitis, three had cryptogenic organizing pneumonia-like and one had ground-glass opacity phenotypes. The patient with grade 4 pneumonitis had a cryptogenic organizing pneumonia-like phenotype and severe radiologic disease. Baseline pulmonary metastatic burden, radiologic findings, and best response to ICI by CTCAE grade are shown in Table 2. Radiologic phenotypes and severity were heterogeneous. Owing to the small number of cases and the absence of imaging for two, no reliable radiologic–clinical outcome correlation could be drawn.

### 3.5. Management and Outcomes

Most patients with grade 1 or 2 pneumonitis were managed in the outpatient setting at diagnosis (7/8, 87.5%), whereas all patients with grade 3 or higher pneumonitis required hospitalization. ICI therapy was held in all 13 patients. All patients with grade 1 pneumonitis initially had ICI therapy held without corticosteroids. Among patients with grade 2 pneumonitis, three received oral corticosteroids and one had ICI therapy held without corticosteroids. Among patients with grade 3 pneumonitis, three received intravenous corticosteroids and one received oral corticosteroids. The patient with grade 4 pneumonitis received intravenous corticosteroids. Nine patients (69%) received systemic corticosteroids at some point, all at 1 mg/kg/day or more of prednisone equivalent, and six (46%) received intravenous corticosteroids.

Pneumonitis improved or resolved in 11 of 13 cases (85%) (Table 3). Two patients, one with grade 1 and one with grade 2 pneumonitis at diagnosis, worsened after initial management, required hospitalization and intravenous corticosteroids, and died 30 and 23 days after diagnosis, respectively; both deaths were classified as grade 5. The patient with grade 1 pneumonitis at diagnosis additionally received infliximab. Pneumonitis was judged the primary cause of death in the patient with grade 2 pneumonitis at diagnosis, who had prior chest radiation and in whom radiation pneumonitis had also been considered; the other death occurred following clinical deterioration attributed to multiple factors, including pneumonitis, infectious pneumonia, and progressive malignancy.

Five patients underwent ICI rechallenge after complete resolution of clinical symptoms; none developed recurrent pneumonitis (0/5; 95% CI 0–52%). Because rechallenge was determined by treating clinicians and restricted to patients who had clinically recovered, this observation is descriptive and should not be interpreted as establishing general rechallenge safety.

## 4. Discussion

In this single-center retrospective cohort of 146 patients with mRCC treated with ICI therapy, pneumonitis occurring during or after ICI treatment was identified in 13 patients (8.9%; 95% CI 4.8–14.7%). This observed incidence exceeds the 4.1% all-grade rate reported for patients with RCC in a meta-analysis of programmed cell death protein 1 (PD-1) inhibitor monotherapy trials [12] and the 3.9% pneumonitis rate reported in a meta-analysis of first-line mRCC clinical trials [13], and ipilimumab plus nivolumab ranked highest for pneumonitis in a network meta-analysis of first-line mRCC regimens [14]. Real-world series have reported higher rates than trials in other diseases; at one institution, 19% of patients with non-small-cell lung cancer treated with PD-1 or programmed death-ligand 1 (PD-L1) inhibitors developed pneumonitis, compared with 3% to 5% in trials [22]. Several features of this cohort probably contributed to the higher rate. More than half of patients received ipilimumab plus nivolumab, and combination blockade carries a higher pneumonitis risk than monotherapy [12]. Fifteen percent had prior chest radiation, and 16% had an ECOG PS of 2 or higher, a group largely excluded from registration trials that required a Karnofsky performance status of 70% or higher [8]. Cases were identified by full record review and multidisciplinary adjudication rather than from investigator-reported adverse events, grade 1 radiographic cases were counted, and onset was not limited to a fixed interval after the last dose. Residual misclassification may also have contributed, because the treating team considered an alternative or contributing cause possible in seven of 13 cases. The confidence interval is wide, and its lower bound (4.8%) lies close to the trial estimates.

This cohort provides information that trial reports and pan-tumor series do not. It is disease-specific: pulmonary metastases are common in mRCC [16], so lung metastases, prior thoracic radiation, and ICI exposure often coexist in the same patient; 82% of this cohort had lung metastases and 15% received chest radiation. Cases were adjudicated by oncologists, pulmonologists, and radiologists, and two thoracic radiologists read each available CT scan independently and blinded to clinical data. Severity was recorded as both grade at diagnosis and highest grade reached, which showed that both deaths occurred in patients who presented with grade 1 or 2 pneumonitis.

The principal exploratory finding was a higher observed incidence of pneumonitis among patients with prior chest radiation. Pneumonitis occurred in 7 of 22 patients (31.8%) with prior chest radiation compared with 6 of 124 patients (4.8%) without prior chest radiation (unadjusted odds ratio 9.2; 95% CI 2.7–31.0). This association should be interpreted cautiously; the width of the confidence interval reflects the small number of events. The analysis was exploratory and limited to univariate comparisons, because 13 events do not support multivariable modeling without overfitting; the association therefore cannot be interpreted as an independent effect of radiation. We could not account for important potential confounders, including radiation dose, fractionation, field, and dose-volume parameters, the interval between radiation and ICI initiation, baseline thoracic tumor burden, ICI agent, and treatment era. This finding is hypothesis-generating and should be evaluated prospectively in larger cohorts with detailed radiation-exposure data.

Prior thoracic radiotherapy has been associated with ICI-related pneumonitis in pan-tumor analyses (pooled adjusted odds ratio 2.07; 95% CI 1.34–3.19) [23] and in lung cancer [24], and pneumonitis after combined anti-PD-1 therapy and radiation has been reported in RCC [25]. Not all data agree: the 2018 American Society of Clinical Oncology (ASCO) guideline cited evidence of no significant difference in irAE rates, including pneumonitis, with thoracic radiotherapy [26]. Biologically, prior radiation may contribute to persistent inflammatory or tissue-injury changes that increase susceptibility to subsequent immune-mediated lung injury [27,28]. Radiation recall pneumonitis is a related clinical entity in patients exposed to checkpoint blockade after thoracic radiation; it can occur months to years after radiotherapy and is typically confined to the previously irradiated lung [29].

Timing argues against classic radiation pneumonitis, which usually appears 1 to 3 months after conventional radiotherapy [29], as the explanation for most irradiated cases in this cohort. Because chest radiation preceded ICI initiation, the interval from radiation to pneumonitis was at least as long as the interval from ICI initiation to pneumonitis, which was 142 days or longer in five of the seven irradiated patients, and onset occurred later in irradiated than in non-irradiated patients (median, 245 vs. 45 days). Radiologists did not have radiation plans, so the current cohort cannot distinguish immune-mediated pneumonitis from radiation recall or determine whether opacities conformed to prior fields. Its findings nonetheless support heightened clinical vigilance when pulmonary symptoms or radiographic abnormalities arise during ICI treatment in patients with prior chest radiation.

We cannot prove an immune mechanism in each case, and the evidence is mixed. Five of 13 patients had another irAE, and 11 improved after ICI was held with or without corticosteroids. Against this, the treating team considered an alternative or contributing cause possible in seven of 13 cases (infection in four, radiation pneumonitis in two, heart failure or volume overload in two, and treatment effect on tumor in one). No histologic finding is specific for ICI-associated pneumonitis, and ASCO considers biopsy generally unnecessary when the clinical picture is consistent [26]; in this cohort, no patient underwent bronchoscopy, and the diagnosis rested on the clinical course and CT findings. Six cases began after ICI had been discontinued, three of them more than 90 days after the last dose (140, 252, and 735 days). Delayed immune-related events, including pulmonary events, have been described a median of 6 months (range, 3–28 months) after discontinuation [21], but attribution becomes less certain as the interval lengthens. When these three cases were reclassified as non-cases, incidence was 6.8% (10/146), and the difference by chest radiation persisted (5/22 vs. 5/124; unadjusted *p* = 0.008). Case-level details are provided in Table S1.

We did not identify statistically significant differences in pneumonitis incidence by smoking history, underlying lung disease, pulmonary metastatic distribution, prior nephrectomy, treatment line, or broad ICI regimen category. These null findings are descriptive and should not be interpreted as evidence that these factors do not influence risk. Smoking history and underlying lung disease have been associated with ICI-related pneumonitis in other settings, particularly non-small-cell lung cancer and extrapulmonary tumor cohorts [23,30]. The small event count and subgroup sizes limited power to detect associations in this mRCC cohort.

Reporting only the grade at diagnosis understated clinical severity. Five of 13 cases (38%) were grade 3 or 4 at diagnosis, but seven (54%) reached grade 3 or higher, including two grade 5 events. Most patients improved or resolved with initial management, but two clinically worsened and died within about a month of diagnosis, both after presenting with grade 1 or 2 pneumonitis; one death was directly attributed to pneumonitis, whereas the other was considered multifactorial. Pneumonitis accounted for 35% of fatal toxic events from PD-1 or PD-L1 inhibitors in the World Health Organization pharmacovigilance database [11]. Onset ranged from 21 to 756 days after ICI initiation (median, 73 days). The median is consistent with the approximately 3 months summarized by ASCO [26], but the range was wider than the 2 to 24 months ASCO reported, and three cases began 9 months or later after ICI initiation (273, 441, and 756 days). These outcomes reinforce the need for close follow-up and rapid reassessment, including evaluation for infection, tumor progression, radiation-associated injury, and immune-mediated pneumonitis, when new respiratory symptoms or pulmonary imaging abnormalities develop during or after ICI therapy, even when the initial presentation is low grade [15,31].

Management after corticosteroid failure rests on retrospective data. Steroid-refractory pneumonitis is commonly defined as no clinical improvement after 48 h of high-dose corticosteroids [18], and ASCO notes that some refractory cases require additional immunosuppression [32]. Agents used in this setting include infliximab, intravenous immunoglobulin (IVIG), mycophenolate mofetil, and cyclophosphamide [18,33,34]. Outcomes with each are poor. In one series, 8 of 12 patients with steroid-refractory pneumonitis died of pneumonitis or infection, including all 5 who received infliximab alone or with IVIG [18]. In another, added immunomodulators, most often tumor necrosis factor-α (TNF-α) inhibitors, produced durable improvement in 10 of 26 patients (38%), and 3 patients died of infections possibly related to immunosuppression [33]. Among patients with lung cancer, pneumonitis improved in 3 of 10 after an added immunosuppressant [35]; in a French pharmacovigilance cohort, 4 of 6 patients treated after steroid failure died within 2 months, and the two survivors had received cyclophosphamide [34]. A systematic review found the most data for mycophenolate and IVIG in steroid-resistant pneumonitis and conflicting data for infliximab [36]. No comparative trial guides the choice between these agents. The one patient in our cohort who received infliximab died after deterioration attributed to pneumonitis, infectious pneumonia, and progressive cancer, so our data cannot inform that choice. For pneumonitis that relapses as corticosteroids are withdrawn, ASCO recommends tapering over at least 4 to 6 weeks [32].

Five patients underwent ICI rechallenge after complete clinical recovery without recurrent pneumonitis (0/5; 95% CI 0–52%). This finding should be considered descriptive only. The upper bound of the confidence interval is compatible with published recurrence rates: the same irAE recurred after 28.8% of informative rechallenges in the World Health Organization pharmacovigilance database [37], and 2 of 7 patients rechallenged with nivolumab developed recurrent pneumonitis in an early series [19]. Rechallenge was selected by treating clinicians and occurred only after clinical recovery in a highly selected subgroup; consequently, these five patients cannot establish the safety of rechallenge for patients with ICI-associated pneumonitis more broadly, and the observation should not be generalized to routine practice.

Radiologic phenotypes were heterogeneous, including COP-like, GGO, hypersensitivity, and nonspecific patterns; the COP-like pattern was the most common, as in earlier descriptions of PD-1 inhibitor pneumonitis [19]. Independent radiology review strengthened the descriptive characterization of available imaging. None of these patterns are specific: in RCC, pulmonary metastases, infection, thromboembolism, heart failure, and TKI-related lung toxicity can produce overlapping symptoms and CT findings [16], and radiation recall produces changes within previously irradiated lung [29]. Imaging was unavailable for two clinically diagnosed cases, and with 11 imaged cases spread across four phenotypes, no reliable radiologic–clinical outcome correlation could be drawn.

This study has several limitations. Its retrospective, single-center design may introduce selection and ascertainment bias; although every record was reviewed in full, ascertainment depended on documentation, and some misclassification is possible. Diagnostic work-up was not standardized, no patient underwent bronchoscopy, and an alternative or contributing cause could not be excluded in seven of 13 cases. Radiation dose, fields, dose-volume parameters, and timing were not available, and radiologists reviewed CT scans without radiation plans. The small number of pneumonitis events limited statistical power, produced wide confidence intervals, and precluded multivariable modeling. ECOG PS was missing for 12 patients, and IMDC risk could not be assigned for 27 because baseline laboratory values were incomplete. ICI start dates spanned 2015 to 2023, with 87% of patients starting on or after April 2018; incidence was similar before and after that date, but the earlier group was small. Prospective multicenter studies with standardized pneumonitis adjudication, detailed radiation data, and prespecified confounder adjustment are needed to validate these observations.

## 5. Conclusions

In this retrospective single-center cohort, pneumonitis occurring during or after ICI therapy was identified in 8.9% (95% CI 4.8–14.7%) of ICI-treated patients with mRCC. Clinical severity and radiologic features were heterogeneous; seven of 13 affected patients reached grade 3 or higher, and two died, both after presenting with grade 1 or 2 pneumonitis. Pneumonitis was more frequent among patients with prior chest radiation in exploratory univariate analysis; this association has a wide confidence interval, could not be adjusted for confounders, and should be evaluated prospectively in larger cohorts with detailed radiation and confounder data.

## Figures and Tables

**Table 1:** Baseline patient characteristics.

	All Patients *n* = 146	Non-Pneumonitis *n* = 133	Pneumonitis *n* = 13
Median Age at Start of Therapy	63.5 years	63 years	66 years
(IQR)	(55–70)	(55–69)	(54–76)
Sex (male)	108 (74%)	98 (74%)	10 (77%)
Race			
White	133 (91%)	121 (91%)	12 (92%)
African American	6 (4%)	5 (4%)	1 (8%)
Hispanic	2 (1%)	2 (2%)	0 (0%)
Asian	2 (1%)	2 (2%)	0 (0%)
Other/Unknown	3 (2%)	3 (2%)	0 (0%)
ECOG PS			
0	39 (27%)	38 (29%)	1 (8%)
1	72 (49%)	61 (46%)	11 (85%)
≥2	23 (16%)	22 (17%)	1 (8%)
N/A	12 (8%)	12 (9%)	0 (0%)
IMDC Risk Group			
Favorable	7 (5%)	6 (5%)	1 (8%)
Intermediate	84 (58%)	73 (55%)	11 (85%)
Poor	28 (19%)	28 (21%)	0 (0%)
Unknown	27 (18%)	26 (20%)	1 (8%)
Histology			
Pure clear cell	107 (73%)	98 (74%)	9 (69%)
Mixed dominant clear cell	9 (6%)	9 (7%)	0 (0%)
Mixed dominant non-clear cell	19 (13%)	15 (11%)	4 (31%)
Unknown	11 (8%)	11 (8%)	0 (0%)
Site of Metastases			
Lung only	17 (12%)	15 (11%)	2 (15%)
Lung + visceral	48 (33%)	44 (33%)	4 (31%)
Lung + soft tissue/nodal	54 (37%)	49 (37%)	5 (38%)
Other than lung	27 (18%)	25 (19%)	2 (15%)
ICI Line of Therapy			
First line	117 (80%)	106 (80%)	11 (85%)
Second line	29 (20%)	27 (20%)	2 (15%)
ICI Regimen			
ICI/ICI	83 (57%)	74 (56%)	9 (69%)
ICI/TKI	20 (14%)	18 (14%)	2 (15%)
ICI monotherapy	43 (29%)	41 (31%)	2 (15%)
Smoking Status			
Never	63 (43%)	55 (41%)	8 (62%)
Former	59 (40%)	55 (41%)	4 (31%)
Current	24 (16%)	23 (17%)	1 (8%)
Underlying Lung Disease (yes)	26 (18%)	23 (17%)	3 (23%)
Cytoreductive Nephrectomy (yes)	82 (56%)	75 (56%)	7 (54%)
Prior Chest Radiation (yes)	22 (15%)	15 (11%)	7 (54%)

Percentages may not sum to 100 because of rounding. ECOG PS, Eastern Cooperative Oncology Group performance status; ICI, immune checkpoint inhibitor; IMDC, International Metastatic RCC Database Consortium; IQR, interquartile range; TKI, tyrosine kinase inhibitor.

**Table 2:** CTCAE grade at diagnosis and corresponding radiographic features of pneumonitis.

CTCAE Grade at Diagnosis	Pneumonitis Phenotype	Radiologic Severity	Lung Metastases Before ICI (no. of Target Lesions)	Lung Target-Lesion iSOM, Mean (mm)	Best Overall Response to ICI
Grade 1 (*n* = 4, 31%)	COP-like (*n* = 2, 50%) GGO (*n* = 1, 25%) Hypersensitivity (*n* = 1, 25%)	Mild (*n* = 2, 50%) Moderate (*n* = 1, 25%) Severe (*n* = 1, 25%)	0 (*n* = 2, 50%) 1 (*n* = 1, 25%) 2 (*n* = 1, 25%)	2.75	iPR (*n* = 1, 25%) iSD (*n* = 1, 25%) iCPD (*n* = 1, 25%) N/A (*n* = 1, 25%)
Grade 2 (*n* = 4, 31%)	Hypersensitivity (*n* = 1, 25%) NOS (*n* = 1, 25%) Not available (*n* = 2, 50%)	Moderate (*n* = 2, 50%) Not available (*n* = 2, 50%)	0 (*n* = 2, 50%) N/A (*n* = 2, 50%)	0 *	iSD (*n* = 1, 25%) N/A (*n* = 3, 75%)
Grade 3 (*n* = 4, 31%)	COP-like (*n* = 3, 75%) GGO (*n* = 1, 25%)	Moderate (*n* = 1, 25%) Severe (*n* = 3, 75%)	1 (*n* = 1, 25%) 2 (*n* = 3, 75%)	25.5	iCR (*n* = 2, 50%) iPR (*n* = 1, 25%) iSD (*n* = 1, 25%)
Grade 4 (*n* = 1, 8%)	COP-like (*n* = 1, 100%)	Severe (*n* = 1, 100%)	0 (*n* = 1, 100%)	0	iCR (*n* = 1, 100%)

Retrospective radiology review of CT scans of patients with pneumonitis was performed by two independent thoracic radiologists blinded to patient clinical data, with consensus review when necessary. Radiologic features were classified into five phenotypic subtypes: cryptogenic organizing pneumonia (COP)-like, ground-glass opacities (GGO), hypersensitivity, interstitial, and pneumonitis not otherwise specified (NOS). Radiologic severity was classified as mild, moderate, or severe. Baseline thoracic metastatic burden was measured qualitatively (number of lung target lesions) and quantitatively (lung target-lesion sum of measurements [iSOM], in millimeters). Best treatment response was evaluated using iRECIST. Metastatic burden, pneumonitis severity and outcome, and best treatment response were determined on serial chest CT scans when available. * Mean of the two patients with imaging available. CTCAE, Common Terminology Criteria for Adverse Events; iCR, complete response; iCPD, confirmed progressive disease; iPR, partial response; iRECIST, immune Response Evaluation Criteria in Solid Tumors; iSD, stable disease; N/A, not available.

**Table 3:** Pneumonitis management and outcomes.

CTCAE Grade at Diagnosis	Initial Pneumonitis Treatment, *n* (%)	Improvement/Resolution with Initial Treatment, *n* (%)	Clinical Worsening with Initial Treatment, *n* (%)	Highest Grade Reached, *n*	Systemic Corticosteroids at Any Time, *n* (%)
Grade 1 (*n* = 4)	ICI hold alone, 4/4 (100%)	3/4 (75%)	1/4 (25%) *	Grade 1: 3 Grade 5: 1	1/4 (25%)
Grade 2 (*n* = 4)	ICI hold alone, 1/4 (25%); oral steroids, 3/4 (75%)	3/4 (75%)	1/4 (25%) *	Grade 2: 3 Grade 5: 1	3/4 (75%)
Grade 3 (*n* = 4)	Oral steroids, 1/4 (25%); intravenous steroids, 3/4 (75%)	4/4 (100%)	0/4 (0%)	Grade 3: 4	4/4 (100%)
Grade 4 (*n* = 1)	Intravenous steroids, 1/1 (100%)	1/1 (100%)	0/1 (0%)	Grade 4: 1	1/1 (100%)
Total (*n* = 13)	Not applicable	11/13 (85%)	2/13 (15%)	Grade ≥ 3: 7 (54%) Grade 5: 2 (15%)	9/13 (69%)

All systemic corticosteroid courses were high dose (1 mg/kg/day or more of prednisone equivalent). ICI, immune checkpoint inhibitor. * Both patients with clinical worsening required hospitalization and intravenous corticosteroids; the patient with grade 1 pneumonitis at diagnosis also received infliximab. Both died and were classified as grade 5. Pneumonitis was the primary cause of death in the patient with grade 2 pneumonitis at diagnosis; the other death was attributed to multiple contributing factors, including pneumonitis, infectious pneumonia, and progressive malignancy.

## Data Availability

The data presented in this study are available on request from the corresponding author. The data are not publicly available because they contain information that could compromise participant privacy.

## Supplementary Materials

The following supporting information can be downloaded at: https://www.mdpi.com/article/10.3390/curroncol33100616/s1, Table S1: Case-level characteristics, management, and outcomes of the 13 patients with pneumonitis; Figure S1: Cumulative incidence of pneumonitis by prior chest radiation.
