# Supplementary material for: Immune Checkpoint Inhibitor-Associated Pneumonitis in Patients Treated for Metastatic Renal Cell Carcinoma
Source: Curr Oncol. 2026 Oct 14;33(10):616. doi: 10.3390/curroncol33100616 (PMC13702867; doi:10.3390/curroncol33100616)
Supplement: Supplementary file 1 [file curroncol-33-00616-s001.zip › curroncol-4555001-supplementary.pdf]

Supplementary Materials

Immune Checkpoint Inhibitor-Associated Pneumonitis in Patients Treated for Metastatic Renal Cell Carcinoma

Table S1. Case-level characteristics, management, and outcomes of the 13 patients with pneumonitis.

Case	CTCAE grade at diagnosis / highest	Onset, days from ICI start	Last ICI dose to onset, days	Prior chest RT	Presenting symptoms	Admitted at diagnosis	Initial management	Systemic CS at any time	Other irAEs	Alternative or contributing cause considered	CT phenotype (severity)	Pneumonitis outcome	ICI rechallenge	Best response to ICI
1	1 / 5	32	On ICI	No	Fever	No	ICI held	IV; infliximab	None	Infection; treatment effect on tumor	COP-like (severe)	Worsened; died, multifactorial	No	N/A
2	1 / 1	68	5	Yes	None	No	ICI held	None	Rash	Radiation pneumonitis	Hypersensitivity (mild)	Improved or resolved	No	iCPD
3	1 / 1	273	252	No	None	No	ICI held	None	Rash, endocrinopathy, arthritis	None recorded	COP-like (moderate)	Improved or resolved	Yes; no recurrence	iPR
4	1 / 1	441	On ICI	Yes	None	No	ICI held	None	None	None recorded	GGO (mild)	Improved or resolved	No	iSD
5	2 / 2	21	On ICI	No	Cough	No	ICI held; oral CS	Oral only	Colitis, endocrinopathy	Infection	Not available	Improved or resolved	Yes; no recurrence	N/A
6	2 / 5	47	5	Yes	Dyspnea, cough	No	ICI held; oral CS	IV	None	Radiation pneumonitis	NOS (moderate)	Worsened; died, pneumonitis primary cause	No	N/A
7	2 / 2	58	On ICI	No	Cough	No	ICI held	None	None	None recorded	Hypersensitivity (moderate)	Improved or resolved	Yes; no recurrence	iSD
8	2 / 2	756	735	Yes	Dyspnea, cough, fever	Yes	ICI held; oral CS	Oral only	Rash, encephalitis	None recorded	Not available	Improved or resolved	No	N/A
9	3 / 3	28	On ICI	No	Dyspnea, cough	Yes	ICI held; IV CS	IV	None	None recorded	COP-like (severe)	Improved or resolved	No	iCR
10	3 / 3	73	On ICI	No	Dyspnea, cough	Yes	ICI held; IV CS	IV	None	Heart failure or volume overload	GGO (severe)	Improved or resolved	Yes; no recurrence	iSD
11	3 / 3	245	140	Yes	Dyspnea, cough	Yes	ICI held; oral CS	Oral only	None	Infection	COP-like (severe)	Improved or resolved	No	iPR
12	3 / 3	256	On ICI	Yes	Dyspnea, cough	Yes	ICI held; IV CS	IV	Arthritis, endocrinopathy	Infection; heart failure or volume overload	COP-like (moderate)	Improved or resolved	Yes; no recurrence	iCR
13	4 / 4	142	16	Yes	Dyspnea, fever	Yes	ICI held; IV CS	IV	None	None recorded	COP-like (severe)	Improved or resolved	No	iCR

Cases are ordered by CTCAE grade at diagnosis and then by onset. Last ICI dose to onset: “On ICI” indicates onset during active ICI treatment; a number gives the days from the last ICI dose to onset. Presenting symptoms are limited to dyspnea, cough, and fever. Systemic CS at any time gives the most intensive route used; all systemic corticosteroid courses were high dose (1 mg/kg/day or more of prednisone equivalent). CT

phenotype and severity are from the independent review by two thoracic radiologists, with consensus review when necessary; imaging was not available for two cases. Best response to ICI was assessed by iRECIST. CS, corticosteroids; COP, cryptogenic organizing pneumonia; CT, computed tomography; CTCAE, Common Terminology Criteria for Adverse Events; GGO, ground-glass opacity; ICI, immune checkpoint inhibitor; iCPD, confirmed progressive disease; iCR, complete response; iPR, partial response; irAE, immune-related adverse event; iSD, stable disease; IV, intravenous; N/A, not available; NOS, not otherwise specified; RT, radiotherapy.

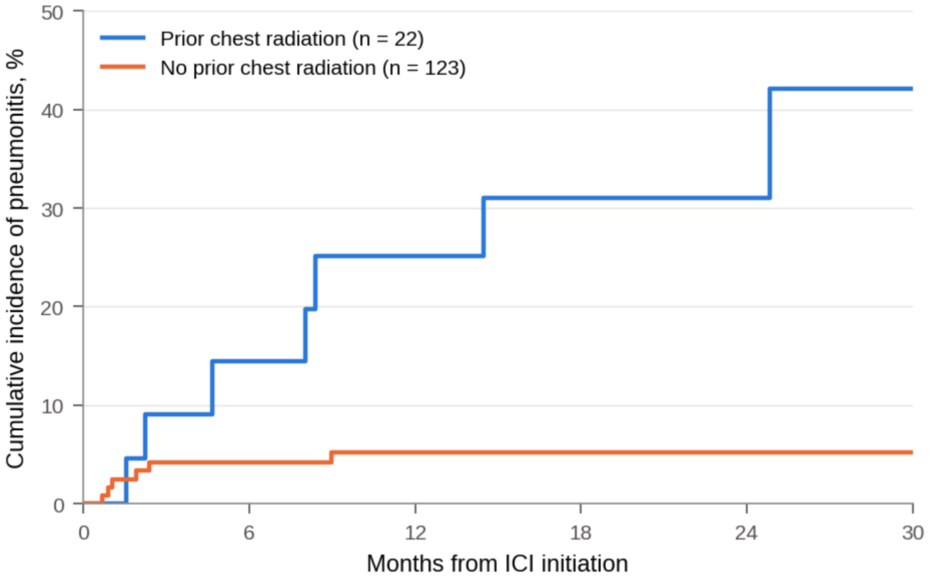

Number at risk

Prior RT	22	15	11	8	4	3
No prior RT	123	86	59	45	26	17

Figure S1. Cumulative incidence of pneumonitis by prior chest radiation. Curves were estimated from ICI initiation with the Aalen–Johansen method, treating death without pneumonitis as a competing risk. At 12 months, cumulative incidence was 25.1% among patients with prior chest radiation and 5.2% among those without. One patient without follow-up time was excluded (n = 145). Numbers at risk are shown below the plot. ICI, immune checkpoint inhibitor; RT, radiotherapy.
